# State Medicine, Public Health, Hygiene and Bacteriology

**Published:** 1895-07

**Authors:** Ernest Wende

**Affiliations:** Health Commissioner of the City of Buffalo, N. Y.


### STATE MEDICINE, PUBLIC HEALTH, HYGIENE AND BACTERIOLOGY.

Conducted by ERNEST WENDE, M. D.,
Health Commissioner of the City of Buffalo, N. Y.

### Synopsis of Recent Measures adopted by the Department of Health toward the Prevention of Consumption.

By William G. Bissell, M. D.,
Bacteriologist, Department of Health, Buffalo, N. Y.

Ever since medicine became a science the contagiousness of consumption has been a mooted question. At different times in its history the opinions of medical men, stimulated, perhaps, by certain superstitions of the laity, have tipped the scale on one side or the other, but never has the question been permanently settled the world over. The day has arrived, however, when there is no doubt as to the contagiousness of the disease. Unfortunately, predisposition is constantly confounded with heredity, even by the profession, but a little thought will clear up the subject to any one who is willing to give it. On the 10th inst., the following circular-letter was mailed to the physicians throughout the city :

DEPARTMENT OF HEALTH.
BUFFALO, N. Y., June 10, 1895.

Dear Doctor—You are aware that tuberculosis is strictly a contagious disease and can be prevented, providing the proper sanitary regulations be adopted. Therefore, after June 15, 1895, the department of health, in the interest of the public safety, demands that all cases of pulmonary tuberculosis (consumption) occurring in your practice be reported to this office, whereupon a circular of instructions will be sent to the family or those in charge of such patients, with the object of lessening and preventing the spread of this dire complaint.

Whenever the diagnosis as to the existence of the disease is in doubt, you are most respectfully requested to submit to the bacteriological bureau of this department the sputum of such cases for a bacteriological test, which will be made free of charge. The report of this examination will be sent you by mail.

Yours most respectfully,

ERNEST WENDE, M. D., *Health Commissioner.*

The following blank of directions accompanied the above circular :

DEPARTMENT OF HEALTH,
BACTERIOLOGICAL LABORATORY,
BUFFALO, N. Y.

*Directions for Collection of Sputum for Bacteriological Examination in Pulmonary Tuberculosis.*

Sputum should be collected only in clean, wide-mouthed, well-stoppered bottles, with a capacity of at least four ounces.

Care should be taken that bronchial and not pharyngeal secretion is collected, and the expectoration discharged early in the morning is to be preferred. If the expectoration is scanty, the entire amount discharged in twenty-four hours should be collected.

As the cases are reported to the department by the attending physician, or found to be tubercular by bacteriological examination, the following circular of instructions is sent to the patient's address, and a full record of the case kept in the department:

DEPARTMENT OF HEALTH,<br>BUFFALO, N. Y.

*Information for Consumptives and Those Living with Them.*

Tuberculosis, commonly known under the names of consumption, decline, scrofula, wasting disease, lupus and white swelling, is a contagious disease, which means that every new case is contracted from some other case. It is not an inherited disease, nor is it due to "a cold," as once supposed, and these facts furnish the key-note of how to prevent the disease. The cause of tuberculosis is a germ, called the bacillus tuberculosis, and the disease is only produced by it. The germ is commonly found in the sputum (spit) of those having consumption and in the pus (matter) discharged from tubercular sores of all kinds. This tubercular germ finds its way into a healthy person, principally in three ways:

1. Through the lungs.
2. Through the stomach and digestive tract.
3. Through open wounds (sores).

1. *Through the Lungs*—This is apt to occur when an ordinary pocket handkerchief is used by a tuberculous person to receive expectoration (spitting)—a filthy and dangerous habit. When such a handkerchief is opened, the dried expectoration becomes pulverized and is disseminated through the air, from whence it may be inhaled by others as well as by the patient himself, who is likely to suffer from drawing the diseased germs into portions of the lung previously unaffected. Another and most common source of pulverized expectoration is derived from the disgusting habit of indiscriminate and careless spitting, as on the floor of street cars, churches, theatres, large stores, public buildings, etc., and on the ground and sidewalks. The expectoration (spit) becomes dry, mixed with dust, and in this form is carried into the air and blown around, then inhaled into the lungs or swallowed. This spitting habit is dangerous in the extreme and should not be practised.

2. *Through the Stomach, etc.*—This occurs generally by consumptives swallowing their own sputum and by the use of spoons, cups and other articles of the kind which have not been properly cleansed after having been used by a tuberculous person or consumptive. Tuberculous meat and milk from tuberculous cows are also a great source of danger and should not under any circumstances be used.

3. *Through Open Wounds* (*cuts*)—This happens by persons getting tubercular pus (matter) into an open cut or an abrasion on the skin, and is probably the least common of the three usual ways of infection.

During the past ten years, Buffalo, with a population of 250,000, has had 5,166 deaths reported as from consumption.

The probabilities are that this is by no means the entire number due to that disease, as certificates are not infrequently falsified in order that relatives may obtain insurance which they otherwise could not were the true cause of death known.

The means of preventing the spread of consumption rests mostly with persons having the disease. If they exercise proper precautions, which are not difficult nor exacting, they can in a great measure avoid giving the disease to others and yet not be deprived of the society of their friends nor of any of the comforts of life.

Knowing the channels of infection, which have been stated, the necessary precautions can easily be taken.

All sputum (spit) from consumptives should be destroyed and must not be allowed to become dry. A spitting cup or flask containing just enough disinfecting solution (which can be made by adding eight drops of carbolic acid to a half a cup of water, or by dissolving a tablet of bichloride of mercury, such as may be procured at any drug store, in a pint of water,) to cover the bottom of the vessel should be used for expectoration. When out of doors a consumptive should use a pocket spitting flask containing the disinfecting fluid. If this is impossible, a piece of old cotton cloth or water-closet paper should be used to spit into, and such cloth or paper destroyed by fire as soon as possible after using. No piece should be used for more than one expectoration.

Never spit on the floors of street cars, public buildings, stores, etc., nor on the ground or sidewalks, as such sputum becomes dried, is blown about and furnishes the source of danger above referred to. There is little danger from the mere breath of a tubercular patient ; the danger lies in the dry expectoration, which contains the contagious or infecting germ.

Kissing consumptives is a positive source of infection, and should be guarded against especially in the case of children. Married people should not sleep together where either are infected. Sleeping in rooms occupied by tuberculous persons is a source of danger, and such rooms should not be used by other persons after having been occupied by consumptives until they have been thoroughly disinfected and all material in them put through the same course. Rooms can practically be disinfected by the use of the fumes of burning sulphur, (using three pounds of sulphur to a room ten feet square and increasing the amount according to the size of the room in proportions of three pounds to each additional 1,000 cubic feet of air space,) the room during the time being tightly closed and allowed to remain so for at least twenty-four hours. The woodwork, walls, wooden parts of bedsteads, chairs, etc., should be washed with a solution of bichloride of mercury (the proportions of which have been given above). For bed springs, etc., a solution of carbolic acid should be used, as mercury will injure the metal.

All dishes, spoons, forks, etc., should be thoroughly washed in boiling water after having been used by a consumptive, and such articles should not be used by any other person in the household until they have been thoroughly boiled.

All meats should be thoroughly cooked and milk sterilized or boiled if it is thought to be from a diseased source.

The bedding and clothing used by a consumptive should not be included in the family wash ; such articles should be washed separately, and be thoroughly boiled during the process of cleansing.

ERNEST WENDE, M. D., *Health Commissioner.*

